# Hetrombopag for the treatment of chemotherapy-induced thrombocytopenia in patients with solid tumors

**DOI:** 10.1016/j.rpth.2025.103326

**Published:** 2025-12-30

**Authors:** Yuan Liu, Bo Liu, Shan-Shan Fang, Run-Sheng Zhao, Lin Li, Hui-Xiong Qi, Quan Li

**Affiliations:** 1 Department of Oncology, Xiangyang Central Hospital, Affiliated Hospital of Hubei University of Arts and Science, Xiangyang, Hubei, China; 2 Department of Gynaecology and Obstetrics, Xiangyang Central Hospital, Affiliated Hospital of Hubei University of Arts and Science, Xiangyang, Hubei, China

**Keywords:** chemotherapy-induced thrombocytopenia, solid tumor, thrombocytopenia, thrombopoietin, thrombopoietin receptor agonists

## Abstract

Based on real-world research, we aimed to systematically evaluate the efficacy and safety of hetrombopag for the treatment of chemotherapy-induced thrombocytopenia (CIT) in patients with solid tumors. Patients with solid tumors who developed CIT (platelet count < 100 × 10^9^/L) and were treated with hetrombopag in a single hospital between February 2022 and September 2023 were included in the study. The primary outcome was complete response rate within 14 days, defined as the proportion of patients with platelet counts of ≥100 × 10^9^/L or platelet counts increased by at least 50 × 10^9^/L from baseline. Response rate within 21 days, the incidence of chemotherapy intensity reduction, the median time of response, and adverse events were reported. A total of 73 patients met the inclusion criteria and were subsequently included in the analysis. The complete response rate within 14 days was 79.5%. Within 21 days, the complete response rate was 91.8%. The incidence of chemotherapy intensity reduction was 21.9%. The median time to platelet response was 9.0 days (95% CI, 8.3-9.7 days). The baseline platelet count of ≥ 50 × 10^9^/L and the treatment regimen of hetrombopag combined with rhTPO/rhIL-11 were identified as independent favorable prognostic factors for platelet response time. Subgroup analyses demonstrated that patients receiving combination regimen exhibited a significantly reduced median time to platelet response with baseline platelet counts of ≥50 × 10^9^/L. Safety profile showed good tolerability of hetrombopag (monotherapy or combined with rhTPO/rhIL-11) in patients. Hetrombopag may be an effective and well-tolerated treatment option for CIT in patients with solid tumors.

## Essentials

• Chemotherapy-induced thrombocytopenia (CIT) is a common problem for patients with solid tumor.
• We retrospectively studied hetrombopag’s efficacy and safety and compared outcomes among different regimens.
• Hetrombopag is an effective and well-tolerated treatment option for CIT in patients with solid tumor.
• Hetrombopag plus rhTPO/rhIL-11 is an independent favorable factor for platelet response time.

## 1 Introduction

Chemotherapy-induced thrombocytopenia (CIT) is one of the most frequent adverse effects associated with antitumor therapies [1,2]. Multiple large-scale retrospective cohort studies have shown that the incidence of CIT ranges from 10% to 21%, with particularly high incidence in regimens involving gemcitabine, carboplatin, and paclitaxel [[3], [4], [5]]. Thrombocytopenia can cause delays in drug administration, dose reductions, interruptions in treatment, bleeding events, and the need for therapeutic platelet transfusions. These complications may compromise the effectiveness of cancer treatments, increase the financial burden of patients, and significantly impact their overall health and well-being [6].

The management of CIT primarily involves platelet transfusion and platelet growth factor therapy. Platelet transfusions can rapidly elevate platelet counts, but they carry risks of transfusion-related adverse events (AEs) such as transfusion reactions, infections, and alloimmunization [7]. Additionally, the effect of transfused platelets is short-lived, making it impractical to maintain adequate platelet levels throughout chemotherapy cycles [8]. Consequently, platelet transfusions are reserved for patients with a high risk of bleeding or severe thrombocytopenia. In comparison, platelet growth factor therapy offers several advantages. Currently, the State Drug Administration has approved recombinant human interleukin (rhIL)-11 and recombinant human thrombopoietin (rhTPO) as first-line treatments for CIT [1]. However, rhIL-11 may lead to complications such as fluid retention, arrhythmias, and pulmonary edema [9], while neutralizing antibodies against rhTPO can cause persistent thrombocytopenia in some patients [10]. These side effects limit their clinical utility. Therefore, there remains an unmet need for safer and more effective therapies in the management of CIT.

In recent years, thrombopoietin receptor agonists (TPO-RAs) have emerged and rapidly progressed into clinical trials, demonstrating promising therapeutic outcomes in CIT [11]. Hetrombopag (Hengqu, developed by Jiangsu Hengrui Medicine Co, Ltd) is an orally administered, small molecule, nonpeptide TPO-RA. It enhances thrombopoiesis through thrombopoietin receptor–dependent signaling pathways, promoting cell viability and preventing apoptosis, thereby exhibiting robust platelet production effects both *in vitro* and *in vivo* [12]. In June 2021, hetrombopag received its first approval in China for treating chronic primary immune thrombocytopenia and severe aplastic anemia [13]. Two clinical studies have shown that hetrombopag is effective and well-tolerated in patients with CIT and solid tumors [14,15]. However, the effectiveness of hetrombopag, along with the comparison between hetrombopag monotherapy and combination regimens, has not been comprehensively evaluated in real-world scenarios.

Therefore, we conducted this retrospective real-world observational study to assess the efficacy and safety of hetrombopag for the treatment of CIT in patients with solid tumors. We then compared the efficacy of hetrombopag when used alone or in combination with rhTPO or rhIL-11 and performed a subgroup analysis.

## 2 Methods

### 2.1 Study design and population

This retrospective observational study was designed to evaluate the efficacy and safety of hetrombopag in the treatment of CIT in patients with solid tumors. The study protocol received approval from the Research Ethics Committee of Xiangyang Central Hospital (approval number 2023-077). The study was reported following the Strengthening the Reporting of Observational Studies in Epidemiology statement.

Patients with solid tumors who developed CIT and were treated at Xiangyang Central Hospital between February 2022 and September 2023 were included in the screening process. The inclusion criteria included the following: (1) age 18 to 80 years old; (2) pathological or cytological diagnosis of a malignant solid tumor requiring antitumor therapy; (3) thrombocytopenia occurred during treatment (platelet count < 100 × 10^9^/L); and (4) received treatment with hetrombopag to elevate the platelet count. The main exclusion criteria were as follows: (1) other causes of thrombocytopenia, such as immune thrombocytopenia, bone marrow invasion, blood disease history, hepatitis, and giant spleen; (2) any known clinically significant acute or active bleeding within 7 days; (3) a history of cardiovascular disease or arterial or venous thrombosis within 3 months; (4) uncontrolled severe complications; (5) pregnant or lactating women; and (6) incomplete follow-up data. Clinical data collected included patient age, sex, tumor type and stage, tumor therapy regimen, platelet enhancing drugs and dosages, adverse drug reactions, comorbidities, blood routine, and liver and kidney function tests. Trained attending physicians were responsible for collecting clinical information related to patients, while 2 senior physicians in the department reviewed inclusion and exclusion criteria and verified patient details and clinical data.

### 2.2 Treatment

All patients received hetrombopag at a dose of 5 to 7.5 mg once daily on an empty stomach. Combination therapy with rhTPO or rhIL-11 for platelet elevation was permitted, with specific dosages as follows: rhIL-11 at 25 to 50 μg/kg administered subcutaneously once daily, and rhTPO at 300 U/kg administered subcutaneously once daily. Treatment was discontinued when platelet counts of ≥ 100 × 10^9^/L or increased by 50 × 10^9^/L from baseline levels. Platelet transfusions were allowed as rescue therapy, following the guidelines and consensus of the Chinese Anti-Cancer Association [1]. Generally, platelet transfusion was indicated if the patient experienced bleeding or had a platelet counts of <10 × 10^9^/L. For patients with World Health Organization grade 1 active bleeding, especially those with necrotic components in their tumors, platelet transfusions were necessary even if the platelet counts were >10 × 10^9^/L to prevent major hemorrhage.

### 2.3 Efficacy outcomes and safety evaluation

The complete platelet response was defined as a platelet count achieving ≥100 × 10^9^/L or increased by at least 50 × 10^9^/L from baseline platelet count. The primary outcome was complete response rate within 14 days from treatment initiation. Secondary outcomes were as follows: (1) complete response rate within 21 days, (2) the incidence of chemotherapy intensity reduction (chemotherapy dose reduction or treatment delay of 4 days or more), (3) the median time from treatment initiation to complete response, and (4) the incidence of platelet transfusion, bleeding, and AEs. AEs were determined according to the Common Terminology Criteria for Adverse Events version 5.0. As the majority of medical records did not include standardized grading descriptions for AEs, no formal severity classification was applied to any reported AE. The incidence and severity of bleeding were recorded and assessed according to the World Health Organization Bleeding scale [16].

### 2.4 Statistical analysis

Categorical variables were presented as numbers and percentages. Continuous variables that follow a normal distribution were reported using means and SDs, while those with nonnormal distributions were described using medians and IQRs. For categorical variables, the chi-squared test or Fisher exact test was used to compare between 2 treatment groups. For continuous variables, the *t*-test or the Wilcoxon rank sum test was used for comparison between groups. The Kaplan–Meier method was used to estimate the median time from the start of treatment to achieving complete response. Univariate and multivariable Cox proportional hazards model analyses were conducted to identify independent factors influencing the platelet response time. Subgroup was analyzed according to baseline platelet count. *P* < .05 was considered statistically significant. All statistical analyses were performed using SPSS version 26.0 (IBM Corp). Graphical representations were created using GraphPad Prism version 9.0.

## 3 Results

### 3.1 Patient characteristics

Between February 2022 and September 2023, a total of 161 patients at our institution met the initial inclusion criteria. Of these, 88 patients were excluded based on the following criteria: 31 patients could not be excluded from having immune thrombocytopenia due to the use of immune checkpoint inhibitors; 5 patients had bone marrow infiltration; 2 patients had cirrhosis with splenomegaly; 33 patients experienced active bleeding within 7 days prior to screening; 15 patients had a history of venous thrombosis within the preceding 3 months; and 2 patient lacked baseline platelet count data. Ultimately, 73 patients were included in the final analysis. Table 1 summarizes the baseline characteristics of the participants. The most common type of cancer was gynecologic tumors, followed by colorectal cancer and lung cancer, which together accounted for over half of all cases. Regarding treatment regimens for thrombocytopenia, 28 patients received hetrombopag monotherapy, whereas 45 patients received combination therapy consisting of hetrombopag with rhTPO (*n* = 41) or rhIL-11 (*n* = 4). There were no significant overall differences in baseline characteristics between the 2 treatment groups, except for a statistically significant difference in baseline platelet count. To facilitate analysis and interpretation, patients who received only 1 treatment regimen in this study were categorized as receiving first-line treatment. Therefore, this classification included not only the initial treatment for advanced or recurrent patients but also adjuvant or neoadjuvant therapies, which together comprised 78.1% of the study population.

**Table 1:** Baseline characteristics of the participants.

Characteristic	Total (*N* = 73)	Treatment group for thrombocytopenia		*P*
		Hetrombopag (*n* = 28)	Hetrombopag-rhTPO/rhIL-11 (*n* = 45)	
Age (y), median (range)	58 (40-76)	58 (40-72)	60 (41-76)	.437
Age (y), *n* (%)				.325
<60	39 (53.4)	17 (60.7)	22 (48.9)	
≥60	34 (46.6)	11 (39.3)	23 (51.1)	
Ethnicity, *n* (%)				1.000
Han Chinese	73 (100.0)	28 (100.0)	45 (100.0)	
Sex, *n* (%)				.859
Male	27 (37.0)	10 (35.7)	17 (37.8)	
Female	46 (63.0)	18 (64.3)	28 (62.2)	
BMI (kg/m^2^), mean ± SD	22.48 ± 3.33	22.30 ± 2.96	22.59 ± 3.57	.724
Cancer type, *n* (%)				.526
Lung cancer	9 (12.3)	4 (14.3)	5 (11.1)	
Gastric cancer	5 (6.8)	2 (7.1)	3 (6.7)	
Colorectal cancer	18 (24.7)	5 (17.9)	13 (28.9)	
Pancreatic cancer	6 (8.2)	1 (3.6)	5 (11.1)	
Cervical cancer	7 (9.6)	2 (7.1)	5 (11.1)	
Ovarian cancer	18 (24.7)	9 (32.1)	9 (20.0)	
Other	10 (13.7)	5 (17.9)	5 (11.1)	
Liver metastases, *n* (%)	22 (30.1)	6 (21.4)	16 (35.6)	.281
Osseous metastases, *n* (%)	3 (4.1)	1 (3.6)	2 (4.4)	1.000
Prior pelvic irradiation, *n* (%)	11 (15.1)	4 (14.3)	7 (15.6)	1.000
Treatment category, *n* (%)				.368
Chemotherapy	52 (71.2)	22 (78.6)	30 (66.7)	
Chemotherapy combined with targeted therapy	21 (28.8)	6 (21.4)	15 (33.3)	
Treatment lines, *n* (%)				.616
1	57 (78.1)	21 (75.0)	36 (80.0)	
≥2	16 (21.9)	7 (25.0)	9 (20.0)	
Baseline platelet count, *n* (%)				.056
≥50 × 10^9^/L	34 (46.6)	17 (60.7)	17 (37.8)	
<50 × 10^9^/L	39 (53.4)	11 (39.3)	28 (62.2)	
Baseline platelet count (×10^9^/L), median (range)	47 (1-85)	67 (3-85)	42 (1-77)	.007
Dose of Hetrombopag (mg), *n* (%)				.448
5	35 (47.9)	15 (53.6)	20 (44.4)	
7.5	38 (52.1)	13 (46.4)	25 (55.6)	

BMI, body mass index; rhIL, recombinant human interleukin; rhTPO, recombinant human thrombopoietin.

### 3.2 Efficacy

The complete response rate within 14 days was 79.5% (58/73), with 53 patients (72.6%) achieving platelet counts of ≥100 × 10^9^/L and 56 patients (76.7%) showing an increase in platelet counts of at least 50 × 10^9^/L from baseline. Within 21 days, the complete response rate was 91.8% (67/73), with 61 patients (83.6%) achieving platelet counts of ≥100 × 10^9^/L and 67 patients (91.8%) had an increase in platelet counts of at least 50 × 10^9^/L from baseline (Table 2). The incidence of chemotherapy intensity reduction was 21.9% (16/73). The median time from treatment initiation to complete response was 9.0 days (95% CI, 8.3-9.7 days).

**Table 2:** Incidence proportion of selected outcomes.

Follow-up (d)	Proportion of platelet count of ≥100 × 10^9^/L, *n* (%)	Proportion of platelet levels increased by 50 × 10^9^/L from baseline, *n* (%)	Complete response rate of any criteria, *n* (%)
14	53 (72.6)	56 (76.7)	58 (79.5)
21	61 (83.6)	67 (91.8)	67 (91.8)

As presented in Table 3, both univariate and multivariable analyses of the platelet response time consistently indicated that patients with baseline platelet counts of ≥50 × 10^9^/L was a key positive predictor of rapid platelet response following hetrombopag treatment. Regarding the treatment regimen, univariate analysis did not reveal a significant difference in response time between hetrombopag combined with rhTPO/rhIL-11 and monotherapy (hazard ratio, 1.113; 95% CI, 0.684-1.811; *P* = .667). Nevertheless, multivariable adjustment revealed that the combination therapy was associated with a significantly faster response and emerged as an independent beneficial factor for shortening platelet response time (hazard ratio, 1.869; 95% CI, 1.084-3.222; *P* = 0.025). In contrast, neither age nor the treatment line had a significant impact on response duration.

**Table 3:** Univariate and multivariable analyses of factors associated with platelet response time.

Variables	Univariate analysis		Multivariable analysis	
	HR (95% CI)	*P*	HR (95% CI)	*P*
Age (y)				
<60	1.211 (0.753-1.948)	.430		
≥60				
Treatment lines				
1	1.185 (0.677-2.077)	.552		
≥2				
Baseline platelet count				
≥50 × 10^9^/L	2.714 (1.652-4.460)	<.001	3.645 (2.077-6.396)	<.001
<50 × 10^9^/L				
Treatment options for thrombocytopenia				
Hetrombopag-rhTPO/rhIL-11	1.113 (0.684-1.811)	.667	1.869 (1.084-3.222)	.025
Hetrombopag				

HR, hazard ratio; rhIL, recombinant human interleukin; rhTPO, recombinant human thrombopoietin.

Given the real-world heterogeneity in treatment protocols, we assessed whether the combination with rhTPO/rhIL-11 influenced efficacy outcomes of hetrombopag. As presented in Table 4, hetrombopag monotherapy group and combination therapy group showed comparable outcomes regarding the 14-day complete response rate, the 21-day complete response rate, the incidence of chemotherapy intensity reduction, and the median time to complete response (all *P* > .05). Considering the inconsistent baseline platelet counts between the 2 treatment groups, we conducted a subgroup analysis with a baseline platelet count of 50 × 10^9^/L as the cutoff point. As shown in Table 5, with respect to platelet response rates at 14 and 21 days as well as the incidence of chemotherapy intensity reduction, no statistically significant differences were observed between the 2 treatment groups, regardless of the baseline platelet count (all *P* > .05). With respect to the median time to platelet response, the outcomes between the 2 treatment groups varied across subgroups. In patients with baseline platelet counts of ≥50 × 10^9^/L, the combination therapy group demonstrated a significantly shorter median response time than the monotherapy group (5 vs 9 days; *P* = .002). In contrast, among those with baseline platelet counts of <50 × 10^9^/L, no significant difference was observed between the two treatment groups (12 vs 11 days; *P* = .683) (Figure).

**Table 4:** Comparison of response outcomes between hetrombopag monotherapy and combination regimens.

Outcome	Hetrombopag (*n* = 28)	Hetrombopag-rhTPO/rhIL-11 (*n* = 45)	*P*
Primary outcome			
14-d response	22 (78.6)	36 (80.0)	.883
Secondary outcome			
21-d response	25 (89.3)	42 (93.3)	.669
Chemotherapy dose reduction/treatment delay	4 (14.3)	12 (26.7)	.214
Median time to response, days (95% CI)	9.0 (8.1-9.9)	9.0 (7.9-10.1)	.640

rhIL, recombinant human interleukin; rhTPO, recombinant human thrombopoietin.

**Table 5:** Subgroup analysis for the response outcomes between hetrombopag monotherapy and combination regimens.

Outcome	Baseline platelet count ≥ 50 × 10^9^/L			Baseline platelet count < 50 × 10^9^/L		
	Hetrombopag (*n* = 17)	Hetrombopag-rhTPO/rhIL-11 (*n* = 17)	*P*	Hetrombopag (*n* = 11)	Hetrombopag-rhTPO/rhIL-11 (*n* = 28)	*P*
14-d response	15 (88.2)	17 (100.0)	0.485	7 (63.6)	19 (67.9)	0.968
21-d response	16 (94.1)	17 (100.0)	0.943	9 (81.8)	25 (89.3)	0.609
Chemotherapy dose reduction/treatment delay	1 (5.9)	0 (0)	0.939	3 (27.3)	12 (42.9)	0.593
Median time to response, days (95% CI)	9.0 (7.8-10.2)	5.0 (4.0-6.0)	0.002	11.0 (4.6-17.4)	12.0 (11.0-13.0)	0.683

rhIL, recombinant human interleukin; rhTPO, recombinant human thrombopoietin.

**Figure 1:**
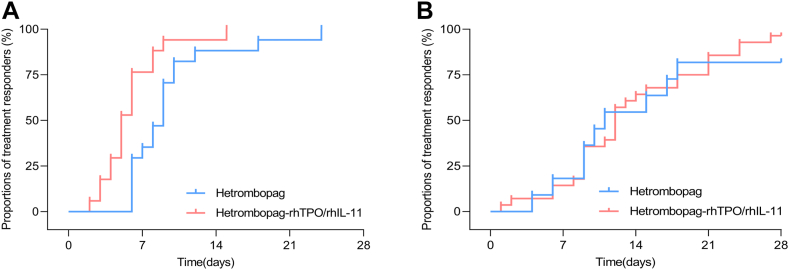
Subgroup analysis of platelet response time between hetrombopag monotherapy and combination regimens. (A) The platelet response time in patients with baseline platelet count of ≥50 × 10^9^/L. (B) The platelet response time in patients with baseline platelet count of <50 × 10^9^/L. rhIL-11, recombinant human interleukin-11; rhTPO, recombinant human thrombopoietin.

### 3.3 Safety

During the observation period, a total of 5 patients experienced bleeding events, with an overall incidence of 6.8%, while 1 patient occurred in the hetrombopag monotherapy group and 4 patients in the combination group. The incidence of platelet transfusion was 16.4% (12/73), with 14.3% (4/28) in the monotherapy group and 17.8% (8/45) in the combination group, respectively. With respect to AEs, the overall incidence was low. Fever was reported in 4 patients (5.5%), with incidences of 7.1% (2/28) and 4.5% (2/45) in the monotherapy and combination groups, respectively. Fatigue occurred in 11 patients (15.1%), with incidences of 14.3% (4/28) and 15.6% (7/45). Decreased appetite was observed in 8 patients (11.0%), with comparable frequencies of 10.7% (3/28) and 11.1% (5/45) across the 2 groups. Peripheral edema developed in 2 patients (2.7%), both received hetrombopag combined with rhIL-11, and this event was not observed in the monotherapy group. Two patients (1.9%) developed deep vein thrombosis in the limbs and were treated with enoxaparin, no further thrombotic events or pulmonary embolisms were observed (Table 6). By September 2025, a total of 11 deaths (15.1%) were recorded among the 73 enrolled patients, comprising 4 patients (14.3%) in the monotherapy group and 7 patients (15.6%) in the combination group. The causes of death included heart failure (1 patient, 1.4%), severe pulmonary infection (1 patient, 1.4%), and tumor metastasis (9 patients, 12.3%), none of which were considered related to the study drug.

**Table 6:** Bleeding, platelet transfusion, and adverse events.

Event	Total (*N* = 73)	Treatment group for thrombocytopenia	
		Hetrombopag (*n* = 28)	Hetrombopag-rhTPO/rhIL-11 (*n* = 45)
Bleeding			
Grade 1	0	0	0
Grade 2	5 (6.8)	1 (3.6)	4 (8.9)
Platelet transfusion	12 (16.4)	4 (14.3)	8 (17.8)
Adverse events			
Fever	4 (5.5)	2 (7.1)	2 (4.5)
Fatigue	11 (15.1)	4 (14.3)	7 (15.6)
Decreased appetite	8 (11.0)	3 (10.7)	5 (11.1)
Peripheral edema	2 (2.7)	0	2 (4.4)
Thromboembolic events	2 (2.7)	0	2 (4.4)

rhIL, recombinant human interleukin; rhTPO, recombinant human thrombopoietin.

## 4 Discussion

The real-world retrospective study results demonstrated that hetrombopag achieved promising efficacy and a favorable safety profile for the treatment of CIT among patients with solid tumors. The baseline platelet count of ≥50 × 10^9^/L and the treatment regimen of hetrombopag combined with rhTPO/rhIL-11 were identified as independent favorable prognostic factors for platelet response time. Subgroup analyses demonstrated that patients receiving combination regimen exhibited a significantly reduced median time to platelet response with baseline platelet counts of ≥50 × 10^9^/L, but no such advantage was observed in the subgroup with baseline platelet counts of <50 × 10^9^/L.

The overall efficacy of hetrombopag observed in this study was consistent with the most evidence for existing TPO-RAs in the treatment of CIT among patients with solid tumors [[17], [18], [19], [20]] and demonstrated sustained therapeutic benefits in a complex real-world population. Specifically, the 14-day complete response rate was 79.5%, rising to 91.8% at day 21. The median time from treatment initiation to complete response was 9.0 days. The incidence of chemotherapy intensity reduction was 21.9%. These outcomes were comparable with a recent phase II randomized controlled trial of hetrombopag, which demonstrated that the proportion of treatment responders (platelet count recovery to ≥100 × 10^9^/L within 14 days) was significantly higher in the hetrombopag group than the placebo group (85.7% vs 48.4%; *P* < .001). The median time to platelet response was 7.5 days in the hetrombopag group, compared with 13 days in the placebo group [14]. The longer median platelet response time observed in our study might be related to the higher proportion of patients with baseline platelet counts of <50 × 10^9^/L. A large multicenter retrospective study evaluated the efficacy of thrombopoietic agents for treating CIT in 1437 patients with solid tumors. The results indicated that the baseline platelet count of <50 × 10^9^/L was independent risk factor for achieving platelet recovery (platelet count ≥ 100 × 10^9^/L) [21], which was consistent with the findings of our Cox proportional hazards model analysis.

The efficacy of hetrombopag remains highly comparable even when compared with that of other TPO-RAs. In a phase II clinical trial of romiplostim for CIT conducted by Soff et al. [19], platelet recovery within 14 days was achieved in 85% (44/52) of patients [19]. While another retrospective analysis of romiplostim treatment revealed that 85% of patients achieved a platelet count of ≥100 × 10^9^/L after initiating therapy, with a median time to response of 9 days. Furthermore, 79% of patients avoided chemotherapy intensity reduction, and 89% did not require platelet transfusion throughout the treatment period [18]. A prospective multicenter, single-arm clinical study of avatrombopag in CIT showed that the cumulative response rate at 21 days was 68.9%, with a median time to platelet recovery of 10.2 ± 6.4 days. Additionally, 18.9% (14/74) of patients required platelet transfusions for rescue therapy [22]. Maintaining chemotherapy intensity is a critical measure of clinical benefit in the current management of CIT. A meta-analysis demonstrated that the thrombopoietic agent group, comprising both first-generation and second-generation agents, had an incidence of chemotherapy intensity reduction of 21.1%, compared with 40.4% in the control group. Although the difference was not statistically significant. Both our study and previous studies also showed that the incidence of chemotherapy intensity reduction was typically approximately 20% after TPO-RAs treatment [15,17,18]. This indicates that TPO-RAs have potential clinical benefits in avoiding the reduction of chemotherapy intensity, but further large-sample, prospective studies are needed to confirm this.

This study further compared the efficacy of hetrombopag monotherapy with that of combination therapy with rhTPO or rhIL-11 in the treatment of CIT in patients with solid tumors. The results showed that, in univariate analysis, there was no significant difference in median response time between the 2 groups. However, combination therapy emerged as an independent favorable factor for shorter response duration after adjusting for baseline platelet count. Moreover, this benefit was observed exclusively in the subgroup with a baseline platelet count of ≥50 × 10^9^/L, whereas no significant difference was observed in patients with a baseline platelet count of <50 × 10^9^/L. Another retrospective study showed that the combination of hetrombopag and rhTPO was significantly more effective than rhTPO alone [15]. Mechanistically, hetrombopag activates the TPO receptor signaling pathway by binding to its transmembrane domain, whereas rhTPO engages the extracellular region. This dual-ligand engagement results in a complementary effect that enhances megakaryocyte proliferation and differentiation in a synergistic manner [6,12]. This multitarget synergy may have mitigated the adverse impact associated with lower baseline platelet counts in the combination treatment group, thereby contributing to an observed response advantage in this study. It is worth emphasizing that the enhanced efficacy observed with the combination was context dependent, varying according to baseline platelet count. This dependency may be associated with the reserve capacity of bone marrow megakaryocytes as reflected by the baseline platelet count [23,24]. The findings in the subgroup analysis suggest a precision stratification strategy for patients with CIT of solid tumors. However, well-designed studies with larger sample sizes are required to confirm these results.

In terms of safety, the most common AEs associated with hetrombopag included upper respiratory tract infections, urinary tract infections, and fever in studies evaluating the treatment of immune thrombocytopenia and severe aplastic anemia [25,26]. Notably, the most frequent hetrombopag-related AEs were elevated platelet counts and increased levels of alanine aminotransferase and aspartate aminotransferase, occurred in ≥5% of patients [13]. In this study, no significant hepatotoxicity was observed, and the AE profile was consistent with findings from phase II clinical trial of hetrombopag for CIT in patients with solid tumors [14]. Overall, hetrombopag demonstrated good safety and tolerability.

There are several limitations to our study that need to be considered. First, this is a retrospective study conducted at a single institution, and the data were collected from medical records. This approach may introduce biases and confounding factors, requiring caution when generalizing the results to a broader CIT population. Second, the nonrandom selection of patients received therapy introduce a selection bias. However, given that hetrombopag is not covered by medical insurance for CIT treatment, clinicians tend to recommend this drug for patients with more severe thrombocytopenia or higher treatment intensity, which may instead lead to an underestimation of its therapeutic effect. Third, as a real-world, it cannot rule out the potential impact of spontaneous platelet count recovery on the outcomes. Finally, the limited sample size and relatively short follow-up period may affect the robustness of the results, particularly in assessing AEs and identifying influence factors.

## 5 Conclusion

In conclusion, our study suggests that hetrombopag may be an effective and well-tolerated treatment option for CIT in patients with solid tumors. The combination of hetrombopag with rhTPO or rhIL-11 has been identified as an independent favorable prognostic factor for platelet response time, particularly in patients with baseline platelet counts of ≥50 × 10^9^/L. However, further randomized controlled trials with larger sample sizes are needed to confirm our findings.
